# Supplementary material for: Decoding and mapping task states of the human brain via deep learning
Source: Hum Brain Mapp. 2019 Dec 9;41(6):1505–19. doi: 10.1002/hbm.24891 (PMC7267978; doi:10.1002/hbm.24891)
Supplement: Supplementary file 1 — Table S1, localization of heatmaps for both GLM and DNN. Table S2. Annotation of in transfer learning to Working Memory Table S3. Annotation of in transfer learning to Motor [file HBM-41-1505-s001.docx]

*S-Table 1, localization of heatmaps for both GLM and DNN.*

|  | **GLM** | | | | | | | **DNN** | | | | | | |  |
| --- | --- | --- | --- | --- | --- | --- | --- | --- | --- | --- | --- | --- | --- | --- | --- |
| **Task** | Annotation | | TalX | TalY | TalZ | Size (mm^2^) | Max Cohen's d | Annotation | | TalX | TalY | TalZ | Size (mm^2^) | Max Cohen's d |  |
| **Emotion** | r | BA.37 | 39 | -58 | -13 | 6120 | 2.49 | r | BA.19 | 43 | -79 | -3 | 5383 | 23.69 |  |
|  | l | BA.19 | -36 | -71 | -10 | 4886 | 2.39 | l | BA.19 | -35 | -77 | -7 | 4487 | 2.47 |  |
| **Gambling** | l | BA.17 | -14 | -89 | 7 | 3718 | 2.79 | l | BA.18 | -4 | -85 | 0 | 6380 | 3.05 |  |
|  | r | BA.17 | 14 | -85 | 10 | 3624 | 2.81 | r | BA.18 | 12 | -81 | -3 | 3607 | 4.37 |  |
|  | r | BA.7 | 29 | -53 | 44 | 454 | 2.24 | l | BA.7 | -37 | -67 | 44 | 1732 | 1.98 |  |
|  | l | BA.7 | -26 | -53 | 43 | 424 | 2.49 |  |  |  |  |  |  |  |  |
| **Language** | l | BA.22 | -52 | -19 | 4 | 1372 | 1.82 | l | BA.22 | -52 | -16 | -6 | 4345 | 3.60 |  |
|  | r | BA.22 | 56 | -13 | 2 | 835 | 1.80 | r | BA.22 | 60 | -14 | 3 | 3327 | 2.74 |  |
|  | r | BA.31 | 11 | -61 | 32 | 700 | -2.14 | r | BA.6 | 48 | 0 | 10 | 440 | 1.64 |  |
|  | l | BA.19 | -12 | -62 | 33 | 673 | -2.30 | l | BA.43 | -60 | -10 | 11 | 422 | 1.88 |  |
|  | r | BA.46 | 37 | 38 | 5 | 425 | -1.52 |  |  |  |  |  |  |  |  |
| **Motor** | l | BA.4 | -36 | -16 | 51 | 3170 | 2.86 | l | BA.4 | -37 | -14 | 52 | 3801 | 3.50 |  |
|  | l | BA.18 | -28 | -92 | -5 | 1072 | -1.88 | r | BA.18 | 10 | -88 | -2 | 3758 | 4.04 |  |
|  | r | BA.18 | 29 | -91 | -1 | 880 | -1.84 | l | BA.19 | -19 | -78 | -2 | 1740 | 2.07 |  |
|  | l | BA.6 | -6 | -3 | 50 | 829 | 2.05 |  |  |  |  |  |  |  |  |
|  | r | BA.6 | 45 | -1 | 42 | 523 | 1.50 |  |  |  |  |  |  |  |  |
|  | r | BA.6 | 7 | 2 | 60 | 489 | 1.50 |  |  |  |  |  |  |  |  |
|  | l | BA.43 | -54 | -18 | 21 | 480 | 1.42 |  |  |  |  |  |  |  |  |
| **Relational** | l | BA.17 | -5 | -82 | 3 | 3134 | 2.83 | l | BA.18 | -12 | -86 | -5 | 6763 | 2.20 |  |
|  | r | BA.18 | 5 | -82 | 2 | 2200 | 2.86 | r | BA.18 | 15 | -85 | -6 | 3144 | 2.23 |  |
|  | l | BA.7 | -26 | -54 | 43 | 1360 | 2.93 | r | BA.19 | 43 | -75 | 21 | 1098 | 2.17 |  |
|  | r | BA.19 | 31 | -61 | 28 | 815 | 2.81 | r | BA.18 | 26 | -71 | 26 | 779 | 1.95 |  |
|  | r | BA.18 | 15 | -94 | 16 | 737 | 2.43 |  |  |  |  |  |  |  |  |
| **Social** | l | BA.19 | -28 | -66 | 23 | 1401 | 3.19 | l | BA.39 | -44 | -68 | 10 | 4982 | 2.80 |  |
|  | r | BA.19 | 40 | -64 | 3 | 1173 | 2.90 | r | BA.19 | 39 | -78 | 24 | 2324 | 7.21 |  |
|  | l | BA.19 | -42 | -63 | 2 | 927 | 3.04 |  |  |  |  |  |  |  |  |
|  | r | BA.7 | 31 | -46 | 48 | 708 | 2.65 |  |  |  |  |  |  |  |  |
|  | r | BA.19 | 29 | -80 | 7 | 691 | 2.82 |  |  |  |  |  |  |  |  |
|  | l | BA.18 | -26 | -84 | 7 | 626 | 2.67 |  |  |  |  |  |  |  |  |
|  | r | BA.19 | 30 | -63 | 25 | 506 | 3.35 |  |  |  |  |  |  |  |  |
| **WM** | r | BA.17 | 16 | -89 | 4 | 4317 | 3.42 | r | BA.19 | 30 | -77 | -3 | 3108 | 2.49 |  |
|  | l | BA.17 | -15 | -94 | 4 | 4155 | 3.30 | r | BA.19 | 39 | -77 | 25 | 1978 | 5.14 |  |
|  |  |  |  |  |  |  |  | l | BA.19 | -19 | -49 | -4 | 1533 | 3.88 |  |
|  |  |  |  |  |  |  |  | l | BA.18 | -8 | -92 | -3 | 413 | 1.85 |  |
|  |  |  |  |  |  |  |  |  |  |  |  |  |  |  |  |

S-Table 2. Annotation of in transfer learning to Working Memory

|  | **GLM** | | | | | | | **CNN** | | | | | | | **SVM-MVPA** | | | | | | |
| --- | --- | --- | --- | --- | --- | --- | --- | --- | --- | --- | --- | --- | --- | --- | --- | --- | --- | --- | --- | --- | --- |
| **Task** | Annotation | | TalX | TalY | TalZ | Size (mm^2^) | Max Cohen's d | Annotation | | TalX | TalY | TalZ | Size (mm^2^) | Max Cohen's d | Annotation | | TalX | TalY | TalZ | Size (mm^2^) | Max F1 Score |
| **0bk**  **body** | r | BA.37 | 45 | -70 | -5 | 5343 | 3.47 | r | BA.19 | 43 | -77 | 1 | 1243 | 1.40 | r | BA.22 | 62 | -7 | 0 | 447 | 74.11 |
|  | l | BA.37 | -42 | -68 | -11 | 2110 | 3.34 | l | BA.19 | -41 | -79 | 0 | 805 | 1.37 |  |  |  |  |  |  |  |
|  | l | BA.17 | -10 | -99 | 8 | 1125 | 2.66 | l | BA.32 | -6 | 20 | -18 | 776 | -1.24 |  |  |  |  |  |  |  |
|  |  |  |  |  |  |  |  | l | BA.18 | -17 | -84 | -5 | 731 | -1.23 |  |  |  |  |  |  |  |
|  |  |  |  |  |  |  |  | l | BA.37 | -28 | -54 | -9 | 512 | -1.10 |  |  |  |  |  |  |  |
| **2bk**  **body** | r | BA.19 | 27 | -81 | -4 | 3893 | 3.57 | l | BA.18 | -10 | -91 | 0 | 3482 | 2.46 | l | BA.9 | -7 | 53 | 27 | 418 | 65.11 |
|  | l | BA.19 | -26 | -80 | -4 | 3516 | 3.71 | r | BA.18 | 14 | -89 | 1 | 2558 | 2.61 |  |  |  |  |  |  |  |
|  | l | BA.7 | -26 | -54 | 42 | 604 | 2.77 | r | BA.3 | 42 | -19 | 46 | 523 | -1.11 |  |  |  |  |  |  |  |
|  |  |  |  |  |  |  |  | l | BA.22 | -57 | -5 | -5 | 406 | -1.36 |  |  |  |  |  |  |  |

S-Table 3. Annotation of in transfer learning to Motor

|  |  | **GLM** | | | | | |  | **CNN** | | | | | |  | **SVM-MVPA** | | | | | |
| --- | --- | --- | --- | --- | --- | --- | --- | --- | --- | --- | --- | --- | --- | --- | --- | --- | --- | --- | --- | --- | --- |
| **Task** | Annotation | | TalX | TalY | TalZ | Size (mm^2^) | Max Cohen's d | Annotation | | TalX | TalY | TalZ | Size (mm^2^) | Max Cohen's d | Annotation | | TalX | TalY | TalZ | Size (mm^2^) | Max F1 Score |
| **left**  **foot** | r | BA.4 | 6 | -19 | 68 | 1920 | 3.18 | r | BA.4 | 6 | -26 | 67 | 3010 | 4.99 | l | BA.4 | 3 | -31 | 65 | 1838 | 74.61 |
|  | l | BA.6 | -9 | 0 | 58 | 960 | 2.41 | l | BA.1 | -46 | -16 | 48 | 1046 | -1.34 | l | BA.1 | 51 | -12 | 33 | 793 | 43.37 |
|  | r | BA.18 | 29 | -92 | -3 | 676 | -1.98 | l | BA.4 | -6 | -12 | 63 | 781 | 1.89 |  |  |  |  |  |  |  |
|  | l | BA.6 | -54 | -1 | 36 | 624 | 1.99 | r | BA.42 | 47 | -36 | 22 | 589 | 1.04 |  |  |  |  |  |  |  |
|  | r | BA.6 | 55 | 1 | 35 | 508 | 2.14 |  |  |  |  |  |  |  |  |  |  |  |  |  |  |
|  | l | BA.18 | -29 | -92 | -6 | 488 | -2.17 |  |  |  |  |  |  |  |  |  |  |  |  |  |  |
| **left**  **hand** | r | BA.4 | 33 | -18 | 44 | 2669 | 3.59 | r | BA.4 | 38 | -14 | 54 | 4631 | 9.48 | r | BA.3 | 36 | -23 | 44 | 2526 | 79.23 |
|  | r | BA.18 | 28 | -92 | -1 | 976 | -2.37 | l | BA.2 | -47 | -23 | 37 | 714 | 1.04 | l | BA.4 | -4 | -30 | 65 | 1870 | 54.25 |
|  | l | BA.18 | -30 | -91 | -4 | 830 | -2.36 | l | BA.4 | -39 | -13 | 57 | 467 | 1.36 |  |  |  |  |  |  |  |
|  | l | BA.4 | -40 | -10 | 55 | 756 | 1.79 | r | BA.19 | 36 | -73 | -7 | 401 | 1.74 |  |  |  |  |  |  |  |
|  | l | BA.6 | -8 | 1 | 59 | 511 | 1.72 |  |  |  |  |  |  |  |  |  |  |  |  |  |  |
|  | r | BA.6 | 7 | -4 | 52 | 463 | 1.72 |  |  |  |  |  |  |  |  |  |  |  |  |  |  |
| **right**  **foot** | l | BA.3 | -5 | -33 | 64 | 2224 | 3.52 | l | BA.4 | -8 | -26 | 66 | 3387 | 6.21 | l | BA.4 | -3 | -33 | 63 | 2282 | 84.58 |
|  | l | BA.40 | -48 | -42 | 24 | 1059 | 2.40 | r | BA.1 | 42 | -22 | 57 | 491 | -1.35 | l | BA.41 | -31 | -25 | 17 | 482 | 44.26 |
|  | l | BA.4 | -43 | -5 | 42 | 905 | 2.08 | r | BA.4 | 40 | -8 | 49 | 438 | 1.37 |  |  |  |  |  |  |  |
|  | l | BA.18 | -29 | -92 | -6 | 620 | -2.31 |  |  |  |  |  |  |  |  |  |  |  |  |  |  |
|  | r | BA.18 | 24 | -96 | -6 | 602 | -2.03 |  |  |  |  |  |  |  |  |  |  |  |  |  |  |
|  | r | BA.6 | 7 | 4 | 60 | 594 | 2.33 |  |  |  |  |  |  |  |  |  |  |  |  |  |  |
| **tongue** | l | BA.4 | -49 | -5 | 30 | 3179 | 4.24 | l | BA.1 | -57 | -8 | 27 | 3641 | 4.52 | l | BA.3 | -46 | -7 | 24 | 2437 | 90.72 |
|  | r | BA.4 | 49 | -4 | 28 | 2308 | 3.43 | r | BA.1 | 58 | -8 | 29 | 2860 | 14.74 | r | BA.3 | 47 | -8 | 24 | 1944 | 86.20 |
|  | l | BA.18 | -24 | -94 | -3 | 603 | -2.30 |  |  |  |  |  |  |  | r | BA.11 | 5 | 34 | -21 | 755 | 59.09 |
|  | r | BA.18 | 20 | -97 | -1 | 575 | -2.25 |  |  |  |  |  |  |  | l | BA.11 | -4 | 43 | -20 | 625 | 53.42 |
|  |  |  |  |  |  |  |  |  |  |  |  |  |  |  | l | BA.24 | -8 | 26 | -12 | 480 | 50.81 |
